# A Dyadic Latent Profile Analysis of Older Couples’ Psychological, Relational, and Physical Health

**DOI:** 10.1093/geroni/igab046.1150

**Published:** 2021-12-17

**Authors:** Josh Novak, Ashley Ermer, Stephanie Wilson

**Affiliations:** 1 Auburn University, Auburn University, Alabama, United States; 2 Montclair State University, Montclair, New Jersey, United States; 3 Southern Methodist University, Dallas, Texas, United States

## Abstract

The present study explored the heterogeneity of older couples’ psychological, relational, and physical health using a sample of 535 couples above the age of 62. A dyadic latent profile analysis was conducted to identify and predict unique clusters of couples’ relative psychological (depressive symptoms and daily hassles), relational (problematic affective communication and marital satisfaction), and physical health (number of health problems and self-reported health satisfaction). Predictors of class membership included relationship length, age, income, and hours worked outside the home. Results revealed 4 distinct classes: Happy & Healthy Together (63.5%), Individually & Relationally Strained (14.7%), Relationally Happy with Strained Wives (12.3%), and Relationally Happy with Strained Husbands (9.3%). Typology descriptions and predictors of class membership will be discussed. These findings highlight that health promotion efforts should be tailored to the specific psychological, relational, and physical health concerns of both partners rather than a one-size-fits-all approach.

